# From policy presence to policy performance: implementation gaps in Philippine food and nutrition systems under systemic shocks

**DOI:** 10.1017/S1368980026102857

**Published:** 2026-06-05

**Authors:** Kervin Blanco Untalan, Genessa Jesy Teroy Pagote, Kyle Renz Gomez Tejero

**Affiliations:** 1 Food Technology, Bukidnon State University, Philippines; 2 Natural Science, Bukidnon State University, Philippines

**Keywords:** Food and nutrition policy, Double burden of malnutrition, Food systems resilience, Policy implementation gap

## Abstract

This commentary examines the gap between the presence of food and nutrition policies and their actual outcomes in the Philippines, with emphasis on how implementation constraints and systemic shocks influence nutrition security. Despite the existence of comprehensive policies and legislative frameworks addressing malnutrition, the country continues to experience persistent stunting, micronutrient deficiencies and increasing overweight and obesity. Rising food prices, energy-related inflation, and economic instability further limit access to nutrient-dense foods, particularly among vulnerable populations. Existing interventions are constrained by fragmented governance, weak targeting mechanisms, limited intersectoral coordination and insufficient adaptive capacity. External shocks further intensify these implementation weaknesses, contributing to what is conceptualised as a ‘shock-amplified implementation gap’. Strengthening governance systems, improving policy implementation and adopting integrated and adaptive food system approaches are essential to achieving sustainable and equitable nutrition outcomes in the Philippines.

## Policy foundations and persistent nutrition challenges

The Philippines has developed a comprehensive legal and policy framework to address food and nutrition security, reflecting a strong institutional commitment to improving population health outcomes. Key legislative measures such as Republic Act No. 11037, Republic Act No. 11148 and Republic Act No. 8976 are complemented by national strategic frameworks such as the Philippine Plan of Action for Nutrition. Collectively, these policies aim to address multiple forms of malnutrition, including undernutrition, micronutrient deficiencies and the growing burden of overweight, obesity and diet-related non-communicable diseases^(1–3)^.

Despite this robust policy architecture, the country continues to face a persistent double burden of malnutrition. Evidence indicates inadequate dietary diversity and insufficient intake of nutrient-dense foods, alongside increasing reliance on low-cost, energy-dense alternatives^(4,5)^. Rising food prices further exacerbate these patterns, driving households to prioritise affordability over nutritional quality^(5,6)^. As a result, undernutrition and overnutrition coexist, reinforcing long-standing nutrition inequalities in low- and middle-income settings^(7)^.

## Current nutrition outcomes and persistent burden

In spite of sustained policy efforts, recent national data indicate that malnutrition and nutrient deficiencies remain significant public health concerns in the Philippines. Findings from the Food and Nutrition Research Institute (FNRI) Expanded National Nutrition Survey show that stunting among children under 5 years of age remains prevalent, alongside persistent micronutrient deficiencies such as Fe deficiency anaemia and vitamin A deficiency among vulnerable populations^(8)^. At the same time, overweight and obesity are increasing among both adults and children, reflecting a dietary shift towards energy-dense but nutrient-poor foods^(7,9)^.

This dual trend underscores the persistence of the double burden of malnutrition, where undernutrition and micronutrient deficiencies coexist with overnutrition and diet-related non-communicable diseases. Similar patterns are observed across low- and middle-income countries, where rising food costs and limited access to healthy diets constrain nutritional adequacy^(4,6)^. In the Philippine context, these challenges are further intensified by economic vulnerability and food price inflation, which limit households’ ability to access diverse and nutrient-rich foods^(10–12)^.

Importantly, these outcomes reflect not only issues of food availability but also persistent constraints in economic access, dietary quality and policy implementation. Evidence shows that rising food prices and income limitations drive households towards cheaper, energy-dense foods at the expense of nutritional quality^(5,6)^, while increased consumption of ultra-processed foods contributes to declining diet quality and adverse health outcomes^(9)^. These patterns suggest that, despite the presence of comprehensive policies, implementation gaps continue to limit their effectiveness in improving population nutrition.

## From policy design to implementation gaps

These persistent outcomes highlight a critical issue: the presence of well-established policies does not necessarily translate into improved population-level outcomes. Rather, they reflect a systemic gap between legislative intent and implementation effectiveness. The central challenge in the Philippine food and nutrition landscape is therefore not policy insufficiency, but policy performance.

The effectiveness of existing laws and programmes is constrained by systemic weaknesses in implementation, including fragmented governance structures, inconsistent budget allocation, weak targeting mechanisms and limited intersectoral coordination. These limitations reduce the capacity of interventions to deliver sustained and equitable nutrition outcomes^(13,14)^.

## External shocks and food system vulnerability

Implementation gaps become more pronounced under conditions of systemic shocks. Food systems in the Philippines are increasingly shaped by external forces such as geopolitical tensions, energy price volatility and macroeconomic instability. In an import-dependent economy, fluctuations in global oil prices directly increase production and distribution costs, contributing to food price inflation^(15,16)^.

Empirical evidence demonstrates that such shocks significantly affect household welfare and food demand, particularly among low-income populations^(17)^. As purchasing power declines, households adjust their consumption patterns, often reducing the intake of nutrient-dense foods in favour of cheaper alternatives, thereby exacerbating nutrition vulnerabilities.

## Policy responses and structural limitations

In response to rising fuel prices and broader economic pressures, the Philippine government has implemented measures such as fuel subsidies for public transport drivers and alternative work arrangements. While these interventions provide short-term relief, their effectiveness is constrained by structural limitations in policy design and implementation.

Evidence suggests that energy subsidies are often fiscally burdensome, poorly targeted and limited in their ability to reach the most vulnerable populations, particularly when not supported by broader structural reforms^(13)^. Macroeconomic analyses further indicate that subsidy-driven interventions, when implemented in isolation, fail to address systemic inefficiencies and may weaken long-term policy effectiveness by diverting resources away from more targeted and sustainable solutions^(9)^.

In the Philippine context, these limitations reduce the capacity of such measures to mitigate the cascading effects of energy price shocks on food systems. Rising fuel costs continue to increase production, transport and distribution expenses, contributing to sustained food price inflation and declining household purchasing power^(15,16,18)^. Consequently, while subsidies may provide temporary relief, they do not sufficiently prevent the deterioration of dietary quality or the persistence of nutrition-related vulnerabilities. This disconnect underscores the role of implementation inefficiencies in shaping food and nutrition insecurity.

## The Shock-Amplified implementation gap

These challenges extend to broader food and nutrition interventions. Programmes such as supplementation, fortification and feeding initiatives, although well established, are often not sufficiently adaptive to rapidly changing economic conditions. Their effectiveness is further constrained by logistical limitations and governance inefficiencies, including issues related to targeting accuracy, delayed delivery, and limited coverage^(13,14)^.

**Figure 1.**
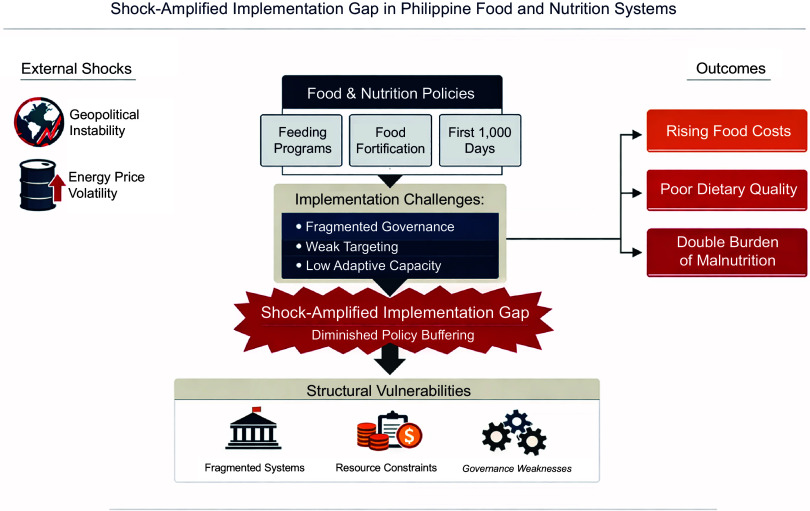
Figure 1 long description. Shock-amplified implementation gap in Philippine food and nutrition systems.

This dynamic can be conceptualised as a shock-amplified implementation gap, wherein external crises expose and intensify underlying weaknesses in governance and public service delivery systems (Figure 1).

## Governance constraints and systemic inefficiencies

Policy responses to recent economic pressures have largely focused on short-term mitigation measures. While these interventions provide immediate support, they do not address the structural drivers of food insecurity and economic vulnerability. Evidence suggests that poorly targeted subsidies may reduce fiscal efficiency and limit long-term impact^(13)^. Without strengthening implementation systems, such measures are unlikely to yield sustainable improvements in nutrition outcomes.

Effective implementation requires strong institutional capacity, clear accountability mechanisms and coordinated action across sectors. However, fragmented institutional arrangements, overlapping mandates and inefficiencies in public financial management continue to hinder policy effectiveness. Weak targeting, resource leakage and limited monitoring systems further reduce the impact of public interventions^(13,14)^.

## Towards integrated and adaptive food systems

Addressing these challenges requires a shift from a policy-centric perspective towards a systems-oriented approach that prioritises implementation capacity and policy coherence. Strengthening governance structures, improving intersectoral coordination and enhancing accountability mechanisms are essential steps towards closing the gap between policy design and policy outcomes.

Equally important is aligning budget allocations with evidence-based priorities and ensuring that interventions effectively reach the most vulnerable populations. Food security must also be addressed within an integrated framework that recognises its interconnections with energy systems, economic stability and global market dynamics. A systems-based approach is therefore critical for building resilience and ensuring sustainable nutrition outcomes^(4,14)^.

## Conclusion

Improving food and nutrition outcomes in the Philippines requires a shift from focusing on policy presence to strengthening policy performance through effective and adaptive implementation. Here, persistent malnutrition does not stem from the absence of policies, but from systemic gaps in governance, coordination and targeting that limit their real-world impact. These constraints are further amplified by economic instability and energy-related shocks, which propagate through food systems and shape household dietary quality, access to food and population health outcomes.

The path forward requires strengthening implementation systems while aligning governance, food, energy and economic policies towards resilience and equity. Policymakers must enhance institutional capacity and ensure that interventions reach the most vulnerable populations. Practitioners must deliver responsive, nutrition-sensitive programmes that address both immediate needs and structural constraints. These efforts must support progress towards the Sustainable Development Goals, particularly SDG 2 (Zero Hunger), SDG 3 (Good Health and Well-being), SDG 7 (Affordable and Clean Energy) and SDG 10 (Reduced Inequalities). Improving nutrition outcomes ultimately depends not only on sound policy design but also on the ability of systems to translate intent into sustained and equitable impact.

## Figure 1. Long description

A diagram illustrating the shock-amplified implementation gap in Philippine food and nutrition systems. The diagram shows the impact of external shocks such as geopolitical instability and energy price volatility on food and nutrition policies. These policies include feeding programs, food fortification, and initiatives focused on the first 1000 days. Implementation challenges such as fragmented governance, weak targeting, and low adaptive capacity are highlighted. These challenges lead to a shock-amplified implementation gap, which results in diminished policy buffering. Structural vulnerabilities including fragmented systems, resource constraints, and governance weaknesses are also depicted. The outcomes of these challenges are rising food costs, poor dietary quality, and the double burden of malnutrition.

Navigate back to Figure 1..
